# HEAR: Approach for Heartbeat Monitoring with Body Movement Compensation by IR-UWB Radar

**DOI:** 10.3390/s18093077

**Published:** 2018-09-13

**Authors:** Wenfeng Yin, Xiuzhu Yang, Lei Li, Lin Zhang, Nattapong Kitsuwan, Eiji Oki

**Affiliations:** 1 School of Information and Communication Engineering, Beijing University of Posts and Telecommunications, Xitucheng Road No.10, Beijing 100876, China; ywf2014@bupt.edu.cn (W.Y.); yangxiuzhu@bupt.edu.cn (X.Y.); 2 School of Computer Science, Beijing University of Posts and Telecommunications, Xitucheng Road No.10, Beijing 100876, China; leili@bupt.edu.cn; 3 Department of Computer and Network Engineering, University of Electro-Communications, 1-5-1 Chofu-shi Chofugaoka, Tokyo 182-8585, Japan; kitsuwan@uec.ac.jp; 4 Graduate School of Informatics, Kyoto University, Yoshida-honmachi, Sakyo-ku, Kyoto 606-8501, Japan; oki@i.kyoto-u.ac.jp

**Keywords:** electrocardiography, ultra wide band radar, variational nonlinear chirp mode decomposition, large body movement compensation

## Abstract

Further applications of impulse radio ultra-wideband radar in mobile health are hindered by the difficulty in extracting such vital signals as heartbeats from moving targets. Although the empirical mode decomposition based method is applied in recovering waveforms of heartbeats and estimating heart rates, the instantaneous heart rate is not achievable. This paper proposes a Heartbeat Estimation And Recovery (HEAR) approach to expand the application to mobile scenarios and extract instantaneous heartbeats. Firstly, the HEAR approach acquires vital signals by mapping maximum echo amplitudes to the fast time delay and compensating large body movements. Secondly, HEAR adopts the variational nonlinear chirp mode decomposition in extracting instantaneous frequencies of heartbeats. Thirdly, HEAR extends the clutter removal method based on the wavelet decomposition with a two-parameter exponential threshold. Compared to heart rates simultaneously collected by electrocardiograms (ECG), HEAR achieves a minimum error rate 4.6% in moving state and 2.25% in resting state. The Bland–Altman analysis verifies the consistency of beat-to-beat intervals in ECG and extracted heartbeat signals with the mean deviation smaller than 0.1 s. It indicates that HEAR is practical in offering clinical diagnoses such as the heart rate variability analysis in mobile monitoring.

## 1. Introduction

Ultra-wideband (UWB) radar has been widely developed for heart rate monitoring, since it provides a contactless measurement. In contrast to the dynamic Electrocardiogram (ECG) monitoring, UWB radar acquires heart rates or respiratory rates without bringing inconveniences in users’ activities in scenarios such as the sleep apnea monitoring. However, it is difficult to sense vital signs of targets with large body movements. A solution [1] for keeping measurements steady chooses to quit detecting when the existence of motion artifacts is recognized. It restricts targets in static states to avoid interferences in extracting vital signs. In order to promote applications of UWB radars in mobile health monitoring, it is necessary to release constraints on targets’ statuses.

To cancel artifacts of large body movements, different strategies are adopted in vital signs monitoring based on the UWB continuous-wave (CW) radar and the impulse radio UWB (IR-UWB) radar. CW radars relieve body movement artifacts by integrating with cameras [2] for phase compensations, implementing self-injection-locked radars [3] or using self and mutual injection locking [4]. In addition, CW radars adopt the empirical mode decomposition (EMD) to cancel sensors’ motion artifacts [5] and applies cross correlations to reduce body movement artifacts [6]. However, CW radars are subject to null-point issues and the small angle approximation. These two issues, which impose limits to single-channel CW architectures, are solved by applying in-phase and quadrature (I/Q) receivers and arctangent demodulations. Special designments, which adopt a single channel mixer to generate a baseband signal linear to vital signals, enable the single-channel CW radar to overcome these two issues as well [7]. In contrast, IR-UWB radar captures vital signs from echos’ time of arrival and thus avoids the null point issue. Therefore, IR-UWB radar is applied in this paper to detect vital signs of moving targets.

IR-UWB radars compensate large body movements by estimating antenna-target distances $\tau_{0}$ and calibrating echos according to $\tau_{0}$. Specifically, in [8], the antenna-target distance $\tau_{0}$ is estimated by locating the time-varying distance with maximum energies along slow time and then fitting with a series of sinusoidal functions. In [9], each echo is calibrated with the first-received echo by the time delay estimation based on cross correlations. After the large body movement is canceled, static-target-oriented methods are applicable to extract heart rates and respiratory rates. The body movement compensation in CW radars is focused on a movement range within 20 cm [3]. The body movement compensation in IR-UWB radars is designed for either large body movements in a range greater than 20 cm [8] or random body movements around a fixed distance [10,11]. In [10], vital signs of a driver are derived by reducing interferences of random body movements of hands, lips or heads. In [11], vital signs of elderly people are detected with canceling random body movements of eating, cooking or sleeping. Contrast to random body movements, body movements of large antenna-target distance changing, such as walking, are compensated in experiments of a virtual thorax motion phantom [9] rather than human targets. It is still challenging to cancel large body movements in real detections of a moving target.

The waveform of heartbeat signals supplies useful information for the heart rate variability analysis in the mobile health monitoring system [12]. Existing methods for extracting heartbeat waveforms include the EMD-based method [13,14] and the variational mode decomposition (VMD) [15,16] based method. EMD-based methods decompose signals into a series of intrinsic mode functions (IMFs), and the IMF whose spectrum is within the heartbeat band is selected as the reconstructed heartbeat signal. The mode mixing issue of EMD causes the coexistence of heartbeats, respiratory harmonics and their intermodulations in one IMF. The frequency resolution of EMD is inadequate to separate close modes. Unlike EMD recursively recovering modes in time domain, VMD simultaneously extracts modes through jointly optimizing in frequency domain. Although VMD gains a better partition of spectrums, it cannot separate wide-band modes with overlapped spectrums. In addition, both EMD and VMD are not capable of obtaining features such as the instantaneous frequency (IF). Since IF affects the beat-to-beat intervals used in the HRV analysis, it is worth trying to acquire precise IF. The variational nonlinear chirp mode decomposition (VNCMD) [17] is capable in decomposing close modes and extracting IF.

**Figure 1:**
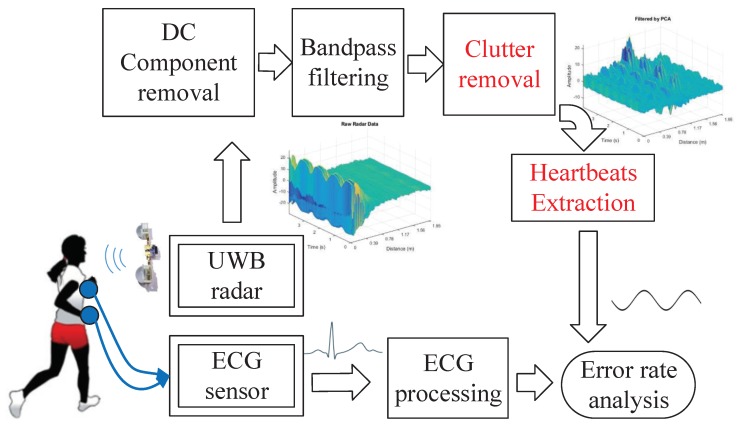
Processing flow of impulse radio-ultra wideband radar data and ECG signal.

This paper puts forward a Heartbeat Estimation And Recovery (HEAR) approach for extracting instantaneous heart rate from a non-stationary target using the IR-UWB radar, as in Figure 1. Firstly, HEAR builds a mapping from amplitude attenuations to antenna-to-target distance variations to compensate large body movements. Based on this mapping, the body movement compensation is achieved by calibrating fast time drifts caused by large body movements. The calibration subtracts the fast time drift estimated by the extended Kalman filter from mapped antenna-to-target distance variations. Secondly, HEAR designs a VNCMD-based heartbeat extraction with optimized initial frequencies. Thirdly, for the clutter removal, HEAR extends the wavelet-based method by designing an exponential threshold with two parameters. The Bland–Altman analysis is carried out to evaluate the consistency of beat-to-beat intervals in ECG and recovered heartbeat signals.

The rest of this paper is organized in the following. Section 2 presents the modification to the wavelet-based clutter removal, states the body movement compensation method and describes the improvement in the VNCMD-based heartbeat extraction. Section 3 presents experimental results and their analyses. Finally, Section 4 concludes our paper.

## 2. Methods

**Figure 2:**
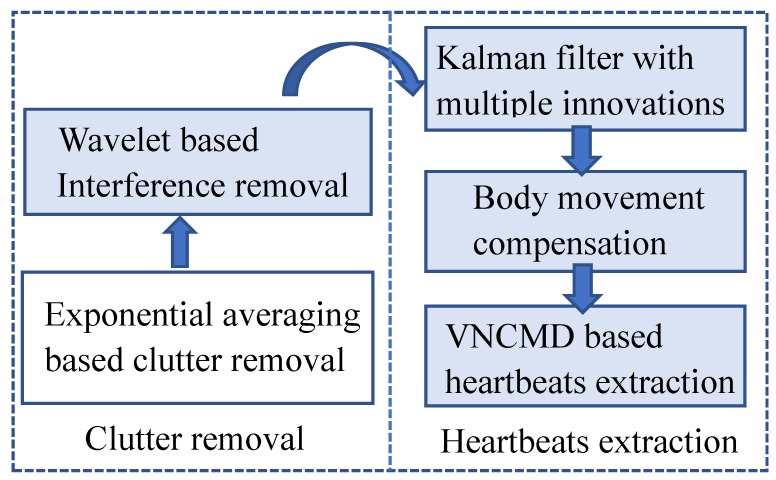
Flow of clutter removal and heartbeat extraction in Heartbeat Estimation and Recovery approach.

HEAR recovers heartbeat signals as procedures in Figure 2. In addition to the clutter removal based on the moving averaging [18], HEAR adopts a wavelet-based method to reduce clutter. HEAR applies a Kalman filter with multiple innovations and observations to denoise the estimations of antenna-to-target distances. A method for cancelling body movements is designed by HEAR according to attenuation characteristics. In order to acquire instantaneous frequencies and waveforms of heartbeats, HEAR extends the VNCMD-based extraction with optimizing initial modal frequencies.

### 2.1. Static Clutter Removal in IR-UWB Radar

Since heartbeat signals are weak body movements, they are easily buried by the static clutter which is the unwanted reflection from surroundings. It is essential to suppress static clutter, caused by the antenna coupling, the impedance mismatch response and the ambient static clutter. Existing algorithms for clutter removal are based on the principal component analysis (PCA) [19], the wavelet [20], the moving averaging and its extensions [18]. Given that the static clutter is similar in different echo signals, the moving averaging algorithm estimates the static clutter by taking a fraction of the previous clutter and a fraction from the current echo signal. In [18], the modeling of static clutter is given by

(1) C(t,τ)=a·C(t−1,τ)+(1−a)·x(t,τ),

where *t* is the slow-time, τ is the fast-time delay, C(t,τ) is the current clutter, x(t,τ) is the raw echo signal, C(t−1,τ) is a previous clutter, and a is the suppressing parameter between [0,1]. If a=1, the current clutter is equal to the previous clutter. If a=0, the current clutter is equal to the raw echo signal.

**Figure 3:**
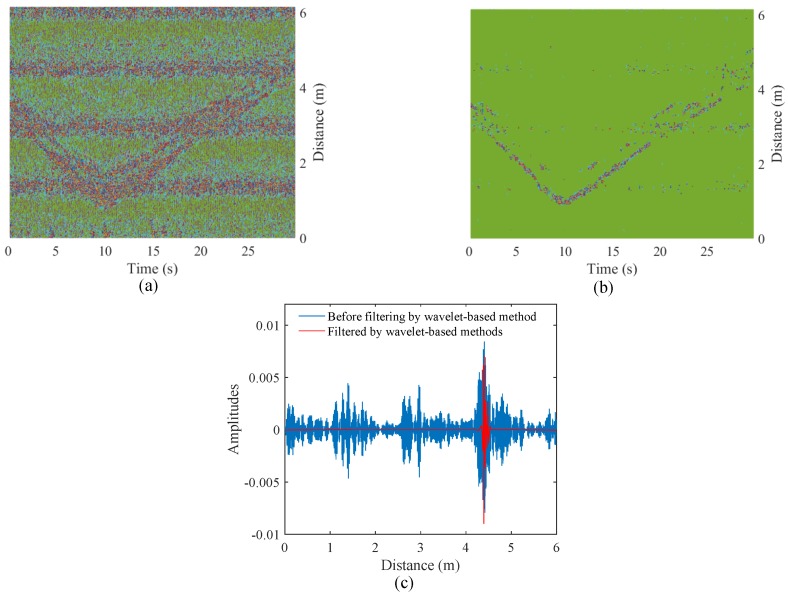
(**a**) Echos filtered by moving averaging methods; (**b**) echos filtered by wavelet-based methods; and (**c**) a specific echo filtered by wavelet-based methods.

Figure 3a illustrates the result of moving averaging with strong static clutter. Since static clutter is preserved after the clutter removal based on moving averaging, HEAR proposes to further reduce interferences by subtracting background echos in multiple levels of wavelet decompositions. Specifically, HEAR adopts an adaptive threshold as

(2) $$thr1\;=\;\;\left\{\begin{array}{cc} \;sign\left(s\left(t,\tau \right)\right)\;*\;\left|\left|s(t,\tau )\right|\;-\;\left|C_{b}\left(t,\tau \right)\right|\right|,\; & \;\left|s(t,\tau )\right|\;>\;\left|C_{b}\left(t,\tau \right)\right|,\\ s(t,\tau )/2, & otherwise, \end{array}\right.$$

where $C_{b}\left(t,\tau \right)$ is the background echos that are collected when there is no target. s(t,τ) is acquired in the scenario with one target. HEAR adopts the biorthogonal wavelet (bior2.6) [21] to decompose echos into 8 levels. Then, thr1 is applied to the first four levels to remove background echos. The biorthogonal wavelet is selected to denoise IR-UWB radar data and meanwhile reduce distortions produced by reconstructions. Since the biorthogonal wavelet is able to solve the contradiction between symmetry and orthogonality requirements, it offers linear phases and assures an accurate signal reconstruction. Reconstruction errors of six kinds of wavelet families are calculated and the least two errors of each wavelet family are listed in Table 1. $Err_{max}$ is the maximum reconstruction error and $Err_{avg}$ is the average reconstruction error. As the comparison shows, $Err_{avg}$ of biorthogonal wavelets is smaller than that of other five categories of wavelets. The energy of bior2.6 is more concentrated in the first four levels than that of bior2.4. For a specific echo, the energy ratio of first four levels to all levels decomposed by bior2.6 is 98.27% while the energy ratio by bior2.4 is 65.85%. Therefore, HEAR chooses bior2.6 to decompose echos into eight levels and performs the threshold-based denoising in the first four levels.

As in [22], the amplitude of arrival clusters follows an exponential decay with the arrival time of clusters and multipaths. HEAR applies an exponential threshold to detailed coefficients at first four levels of wavelet decompositions as

(3) $$\mathrm{thr}2(y+x)=ae^{-bx/N}e^{-2cy/M},$$

where *y* is the arrival time of clusters and *x* is the arrival time of multipaths. *N* is the multipath number in a cluster and *M* is the total multipath number in all clusters. *b* and *c* are respectively the attenuation factors of multipaths and clusters. *a* is the proportion factor of echos to unit channel models. HEAR determines *N* by taking a cluster as 0.5 m. *c* is calculated as the ratio of the maximum to the average of an echo. *b* is set as 0.5. thr2 is applied to the first four levels to reduce multipath interferences. After background echos and multipath interferences are removed, the inverse wavelet transform is carried out to recover target echos. Figure 3b depicts echos with static clutter subtracted by the wavelet-based method. As the specific filtered echo in Figure 3c shows, multipath interferences are reduced effectively.

### 2.2. Body Movement Compensation

As stated in [23,24], a time-varying channel impulse response is set as

(4) $$\begin{array}{cc} h(t,\tau ) & =h_{static}\left(t,\tau \right)+h_{mobile}\left(t,\tau \right)\\ & =\sum_{i=1}^{M}\alpha_{i}\delta \left(\tau -\tau_{i}\right)+\sum_{j=1}^{N}\alpha_{j}\delta \left(\tau -\tau_{j}\left(t\right)\right), \end{array}$$

where *M* denotes the number of channel responses from static backgrounds, *N* denotes the number of channel responses from the target, *t* is slow-time, τ is fast-time, $\tau_{i}$ and $\tau_{j}$ represent the reflection delay, $\alpha_{i}$ and $\alpha_{j}$ are amplitudes of the channel response. The received echo signal is obtained as a convolution of the channel response and the transmitted impulse p(τ), as

(5) $$\begin{array}{cc} s(t,\tau ) & =p(\tau )*h(t,\tau )\\ & =\sum_{i=1}^{M}\alpha_{i}p\left(\tau -\tau_{i}\right)+\sum_{j=1}^{N}\alpha_{j}p\left(\tau -\tau_{j}\left(t\right)\right). \end{array}$$

According to [25], the transmitted impulse is a mixture of derivatives of Gaussian impulse, as $p\left(\tau \right)=\sum_{i}b_{i}e^{-\frac{2\pi {\left(\tau -\tau_{i}\right)}^{2}}{\beta^{2}}}$. The formation parameter $\beta^{2}\;=\;4\pi \sigma^{2}$ and $\sigma^{2}$ is the variance of Gaussian pulses. It is supposed that the response of static channel is removed by the clutter removal, i.e., $\sum_{i=1}^{M}\alpha_{i}p\left(\tau -\tau_{i}\right)=0$. Therefore, the received echo signal is formulated as

(6) $$s\left(t,\tau_{a}\right)=\sum_{j=1}^{N}\alpha_{j}p\left(\tau -\tau_{j}\left(t\right)\right)=\sum_{j=1}^{N-1}s_{j}+s_{c},$$

where $s_{c}=A_{c}e^{-\frac{4\pi {\left(\tau -\tau_{c}\left(t\right)\right)}^{2}}{\beta^{2}}}$. $A_{c}$ is the amplitude after impulses pass through the channel and $\tau_{c}\left(t\right)$ is the vibration of chest. s(t,τ) contains series of sub-echos $s_{j}$ reflected from different positions of the target, including the specific echo $s_{c}$ from the chest.

According to the above modeling, it is feasible to extract the vital signal from the changing fast-time delay related to maximum echo amplitudes. In Equation (5), when s(t,τ) reaches its maximum at the slow time $t_{i}$, the corresponding fast-time delay is denoted as $\tau_{a}$. The HEAR approach treats the $\tau_{a}$ as a weighted sum of the reflection delay $\tau_{j}$ of sub-echos, as

(7) $$\tau_{a}\left(t_{i}\right)=\sum_{j=1}^{N-1}w_{j}\tau_{j}\left(t_{i}\right)+w_{c}\tau_{c}\left(t_{i}\right),$$

where $w_{j}$ and $w_{c}$ are weights related to the energy ratio of each sub-echo to the mixed echo, $w_{c}$ is the weight of sub-echos from the chest, and $\sum_{j=1}^{N-1}w_{j}+w_{c}=1$. As in [26], the vibration of chest $\tau_{c}\left(t\right)$ is modeled as a sum of the antenna-to-target distance $\tau_{d}\left(t\right)$ and the vital signal $\tau_{v}\left(t\right)$ containing harmonics of respiration and heart beating, as

(8) $$\begin{array}{cc} \tau_{c}\left(t\right) & =\tau_{d}\left(t\right)+\tau_{v}\left(t\right)\\ & =\tau_{d}\left(t\right)+\sum_{p}\tau_{b}^{p}\sin\left(pf_{b}t+\phi_{b}\right)+\sum_{q}\tau_{h}^{q}\sin\left(qf_{h}t+\phi_{h}\right), \end{array}$$

where *p* and *q* denote the harmonic order, $f_{b}$ and $f_{h}$ are respectively the respiratory frequency and the heartbeat frequency, and $\phi_{b}$ and $\phi_{h}$ are respectively initial phases of respiration and heartbeat.

Vital signals are generated by body micromovements such as breathing and heart beating. $\tau_{d}\left(t\right)$ changes as target’s large body movements including walking or body shifting of a sitting target. In order to reduce interferences of large body movements, HEAR estimates body micromovements by amplitude attenuations and then compensating large body movements $\tau_{d}$ by an extended Kalman filter.

• Large body movement estimation

Large body movements are estimated by adopting a distance estimation method which integrates the multi-innovation Kalman (MIK) filter and the k-Nearest Neighbor. MIK filter applies multiple innovations of previous moments to predict antenna-to-target distances, achieving smoother estimations than conventional Kalman filters. MIK updates distance estimations as [27]

(9) $${\hat{x}}_{k}={\hat{x}}_{k|k-1}+\sum_{i=1}^{p}K_{i}\left(k\right)\left(z_{k-i+1}-C_{k-i+1}{\hat{x}}_{k-i+1|k-i}\right),$$

where ${\hat{x}}_{k}$ is the updated distance estimation at current time *k*. ${\hat{x}}_{k|k-1}$ is the posteriori distance estimation at time *k*. $z_{k}$ is the observation of antenna-to-target distances at time *k*. $K_{i}$ is the optimal Kalman gain. $C_{k}$ is the noise covariance matrix. *p* is the number of previous innovations used for current predictions.

**Figure 4:**
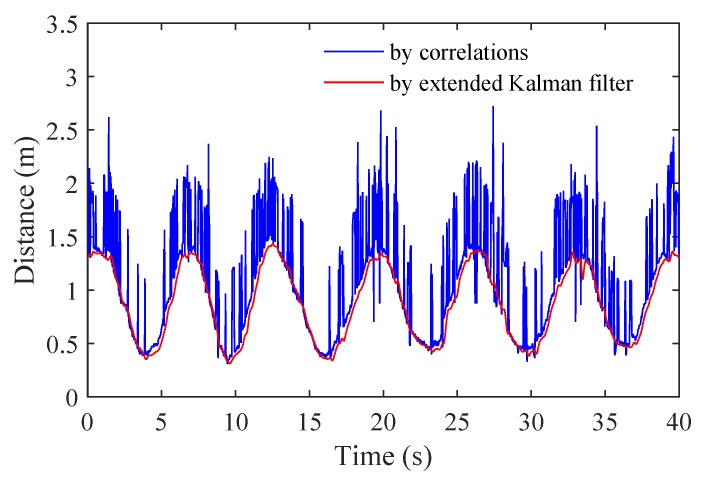
Large body movements $\tau_{a}$ estimated by two approaches.

Because of multipath interferences, there are multiple observations of distances for one target at time *k*. HEAR combines the MIK filter with KNN to avoid deviations imported by selecting one distance from all observations as the input of MIK filtering. Specifically, during each iteration of MIK filtering, KNN is employed to select out the distance value $z_{k}$ which is most close to previous *p* estimations of distances $\left\{{\hat{x}}_{k-1},\cdots ,{\hat{x}}_{k-p}\right\}$. HEAR applies the extended MIK filtering to predict the fast time delay $\tau_{a}$ varying as large body movements. Figure 4 illustrates large body movements $\tau_{a}$ estimated by the extended Kalman filter and the correlation-based approach.

• Body micromovement estimation

Existing methods recover heartbeat waveforms by decomposing echo amplitudes with EMD, which depends on maximums and minimums of amplitudes. Body movements enlarge multipath interferences in echo amplitudes and therefore cause distortions in heartbeat waveforms reconstructed by EMD. To reduce interferences of body movements in recovering heartbeat waveforms, HEAR transfers amplitude variations into relative fluctuations of the fast time delay.

**Figure 5:**
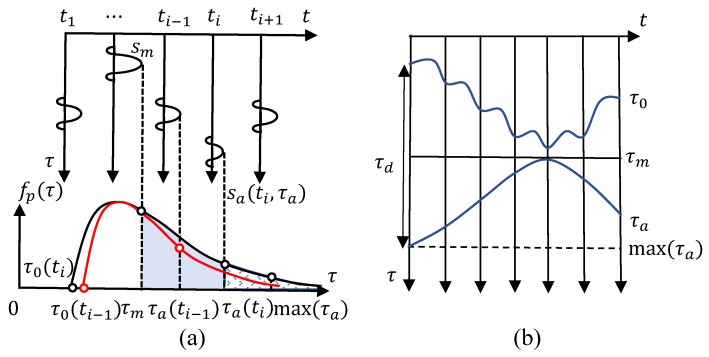
(**a**) mapping between $s_{a}$ and $\tau_{0}$; (**b**) relations between $\tau_{0}$ and $\tau_{d}$.

Supposing the echo amplitude s(t,τ) reaches its maximum $s_{m}$ at $\tau_{m}$ along the slow time, amplitude attenuations compared to $s_{m}$ are proportional to increments of the fast time delay compared to $\tau_{m}$. It is assumed that the channel fading follows a Rayleigh distribution. The attenuation ratio of a specific amplitude $s_{a}$ to $s_{m}$ is equivalent to the ratio of their amplitudes appearing possibilities $P(\tau \geq \tau_{a})$ and $P(\tau \geq \tau_{m})$. As shown in Figure 5a, the difference of $\tau_{0}$ and $max(\tau_{a})$ is determined by the attenuation ratio of $s_{a}$ to $s_{m}$. Given that $max(\tau_{a})$ and $s_{m}$ are constants, $\tau_{0}$ varies corresponding to variations of $s_{a}$. As to the instance in Figure 5a, the attenuation ratio of $s_{a}$ to $s_{m}$ at time $t_{i}$ is larger than that at $t_{i-1}$ marked in the red line, and the difference $max\left(\tau_{a}\right)-\tau_{0}$ at $t_{i}$ is greater than that at $t_{i-1}$. Specifically, the transform from amplitudes $s_{a}$ to the fast time delay $\tau_{0}$ is calculated by solving the following equation:

(10) $$\frac{s_{a}\left(t_{i},\tau_{a}\right)}{s_{m}\left(t_{m},\tau_{m}\right)}=\frac{P(\tau \geq \tau_{a})}{P(\tau \geq \tau_{m})}=\frac{e^{-{\left(\tau_{a}+\tau_{0}\left(t_{i}\right)\right)}^{2}/2\sigma^{2}}}{e^{-{\left(\tau_{m}+\tau_{0}\left(t_{i}\right)\right)}^{2}/2\sigma^{2}}},$$

where the variance σ is set as $\frac{1}{3}\left[max\left(\tau_{a}\right)-\left(\tau_{0}\right)\right]$. The probability of amplitudes attenuation is $P\left(\tau \geq \tau_{a}\right)=e^{-\tau^{2}/2\sigma^{2}}$. After acquiring $\tau_{0}\left(t_{i}\right)$ from Equation (10), the distance $\tau_{d}\left(t_{i}\right)$ is obtained as $max\left(\tau_{a}\right)-\tau_{0}\left(t_{i}\right)$. Figure 5b shows the relation between $\tau_{0}$ and $\tau_{d}$.

**Figure 6:**
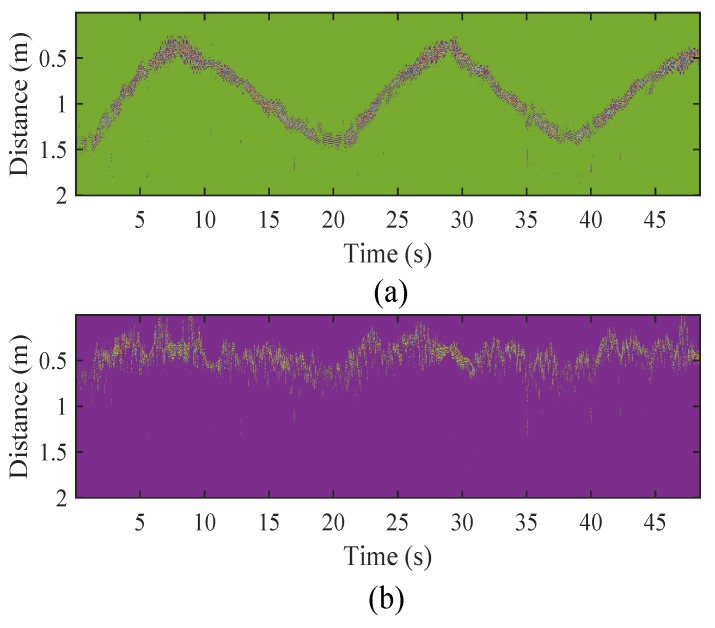
Echos (**a**) before and (**b**) after body movements’ compensations.

Figure 6b illustrates the aligned echos after compensating large body movements in Figure 6a. The large body movement is compensated by subtracting $\tau_{d}$ from $\tau_{a}$. The vital signal $\tau_{v}\left(t\right)$ is contained in the subtraction between $\tau_{a}$ and $\tau_{d}$ as in Equation (11), and therefore it is extractable by applying VNCMD in the following procedure. Algorithm 1 describes specific procedures for capturing the vital signal $\tau_{v}\left(t\right)$:

(11) $$\tau_{a}\left(t\right)-\tau_{d}\left(t\right)=\sum_{j=1}^{N-1}w_{j}[\tau_{j}\left(t\right)-\tau_{d}\left(t\right)]+w_{c}\tau_{v}\left(t\right).$$

After mapping maximum amplitudes to the fast time delay, large body movement compensations become feasible by a simple subtraction. In addition, waveform distortions of vital signs caused by large body movements are relieved.

**Algorithm 1:** Flow of extracting vital signal $\tau_{v}\left(t\right)$. **Input:** Filtered echo matrix $s(t_{i},\tau_{j})$, 1≤i≤L, 1≤i≤K.
 **Output:** Chirp signal $g(t_{i})$ containing the vital signal $\tau_{v}\left(t_{i}\right)$, 1≤i≤L.
  1: Along the ridgeline of $s(t_{i},\tau_{j})$, save the amplitudes in to a vector $s_{a}\left(t_{i}\right)$, 1≤i≤L;
  2: Along the ridgeline of $s(t_{i},\tau_{j})$, save the fast time delay in to a vector $\tau_{a}\left(t_{i}\right)$, 1≤i≤L;
  3: Find the maximum in $s(t_{i},\tau_{j})$, note the amplitude as $s_{m}$, and save the fast time delay as $\tau_{m}$;
  4: Obtain the relative fast-time delay $\tau_{d}\left(t_{i}\right)$ as $max\left(\tau_{a}\left(t_{i}\right)\right)-\tau_{0}\left(t_{i}\right)$ by solving Equation (10);  5: Compensate large body movements as Equation (11);  6: **return**
$g(t_{i})$;

### 2.3. Separation of Heartbeats and Respirations by VNCMD

EMD is a method more general than filtering methods for separating heartbeats and respirations. However, the mode mixing problem in EMD hinders separating heartbeats from those respiratory harmonics whose energies are greater than heartbeats’ in the heartbeat band [1 Hz, 2 Hz]. VMD is an adaptive decomposition method which suppresses mode mixing problems in EMD. As in [17], VNCMD overcomes limitations that VMD cannot acquire instantaneous amplitudes and instantaneous frequencies from each mode. In order to obtain instantaneous heart rates and heartbeat waveforms, the HEAR approach adopts the VNCMD to extract heartbeat signals.

As in Equation (11), the vital signal is contained in a chirp signal g(t), which is equivalent to

(12) $$\begin{array}{cc} g(t) & =\tau_{a}\left(t\right)-\tau_{d}\left(t\right)\\ & =\sum_{p}\tau_{b}^{p}\sin\left(pf_{b}t+\phi_{b}\right)+\sum_{q}\tau_{h}^{q}\sin\left(qf_{h}t+\phi_{h}\right)\\ & +\sum_{j}\tau_{b+h}^{j}\sin\left(\left(nf_{h}+kf_{b}\right)t+\phi_{b+h}\right)+n\left(t\right), \end{array}$$

where *j*, $\tau_{b+h}^{j}$ and $\phi_{b+h}$ are respectively the number, the fast time delay and the initial phase of intermodulations of respirations and heartbeats. The intermodulations are imported to the fast time delay $\tau_{v}$ by the mapping from amplitudes, since the amplitude is regulated by the intermodulations as well. Specifically, the echo amplitude $s_{c}$ in Equation (6) is expanded by the Taylor series as

(13) $$\begin{array}{cc} s_{c} & =e^{-{\left(\Delta \tau -\sin\left(f_{b}t\right)-\sin\left(f_{h}t\right)\right)}^{2}}\\ & =1+\sum_{n=1}^{\infty }\frac{1}{n!}{\left(\Delta \tau -\sin\left(f_{b}t\right)-\sin\left(f_{h}t\right)\right)}^{2n}\\ & =1+\sum_{n=1}^{\infty }\frac{1}{n!}{\left(\Delta \tau^{2}-2\Delta \tau \sin\left(f_{b}t\right)-2\Delta \tau \sin\left(f_{h}t\right)+h(t)\right)}^{n} \end{array}$$

(14) $$\begin{array}{cc} h(t)= & 1-\cos\left(\left(f_{b}+f_{h}\right)t\right)+\cos\left(\left(f_{b}-f_{h}\right)t\right)\\ & \;-\frac{1}{2}\cos\left(2f_{b}t\right)-\frac{1}{2}\cos\left(2f_{h}t\right), \end{array}$$

where $\Delta \tau =\tau_{a}-\tau_{d}$. To be simplified, the coefficients of Gaussian impulses are omitted and no harmonic of respirations and heartbeats is considered in Equation (13). Therefore, the chirp signal g(t) comprises multiple frequencies including respiration rate $f_{b}$, heartbeat rate $f_{h}$, their harmonics and intermodulations. VNCMD is capable of recovering the time-varying heartbeat signal as a nonlinear chirp mode (NCM) decomposed from g(t). VNCMD requires initial frequencies for each NCM to be recovered. It is feasible to initialize modal frequencies by extracting ridge curves of the short time Fourier transform (STFT).

As discussed in [17], the convergence of VNCMD depends on the noise level and the specified initial modal frequencies. A good initialization of modal frequencies helps VNCMD in converging in noisy situations. If the relative error between initial frequencies and real frequencies is smaller than 60%, the VNCMD still has a great success rate to converge to correct modal frequencies even at a strong noise level. To ensure that the VNCMD converges at the respiratory frequency and the heartbeat frequency rather than their harmonics, the initial modal frequency should contain frequency estimations of respirations and heartbeats. Since respiratory harmonics are stronger than heartbeats in spectrums in some cases, it is not sure that the frequency extracted by STFT in the heartbeat band is the heartbeat frequency. It is necessary to optimize the initial modal frequencies to prevent them from losing the respiratory frequency and the heartbeat frequency. HEAR recovers the initial frequencies of respirations and heartbeats by solving the following quadratic programming issue:

(15) $$\min\sum_{i=1}^{3}f_{i}-nf_{h}-kf_{b}-\left(mf_{h}+tf_{b}\right),$$

(16) $$\begin{array}{ll} s.t. & kf_{b}=f_{1},\\ & nf_{h}=f_{2},\\ & mf_{h}+tf_{b}=f_{3}, \end{array}$$

where $f_{i}$ is the frequency extracted on ridges in STFT of the vital signal. $f_{b}$ is the respiratory frequency and $f_{h}$ is the heartbeat frequency. *n*, *k*, *m* and *t* are respectively orders of respiratory harmonics and heartbeat harmonics. The lower bound for the vector $x=\{f_{b},f_{h},n,k,m,t\}$ to be optimized is set as [0.01,1,0.5,0.7,−5,0.7], and the upper bound is set as [0.8,2.5,4,1.5,5,1.5].

**Figure 7:**
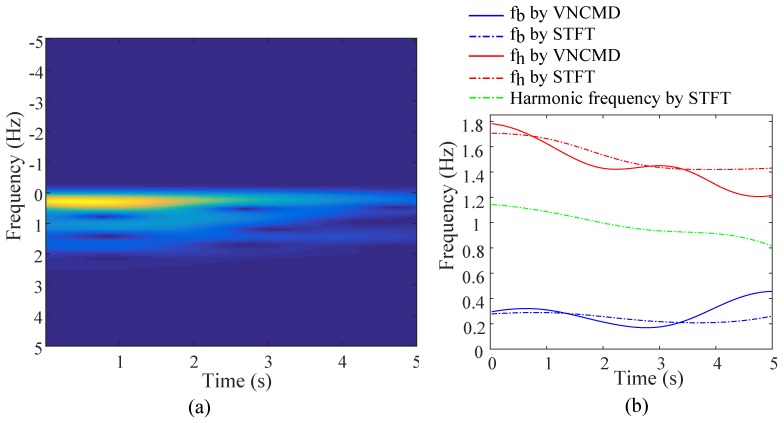
(**a**) Results of short time Fourier transform (STFT), and (**b**) extracted frequencies by STFT and variational nonlinear chirp mode decomposition (VNCMD).

The above optimization assumes that all frequencies extracted from STFT are composed of *k*th respiratory harmonics $kf_{b}$, *n*th heartbeat harmonics $nf_{h}$ and their intermodulations $mf_{h}+tf_{b}$. The optimization poses constraints on both harmonic orders and frequencies of respirations and heartbeats. Specifically, values of respiratory harmonics, heartbeat harmonics and their intermodulations are restricted as respectively equal to an extracted frequency in $\left\{f_{i}\right\}$, as described in formula (16). The optimization is achieved by reducing each discrepancy between the harmonic and the corresponding extracted frequency in $\left\{f_{i}\right\}$. Figure 7b illustrates frequencies derived from the STFT in Figure 7a and estimated by VNCMD.

## 3. Experiments

In experiments, ECG recordings are collected simultaneously with IR-UWB radar data and further processed to extract beat-to-beat intervals as references.

### 3.1. Experimental Settings

**Figure 8:**
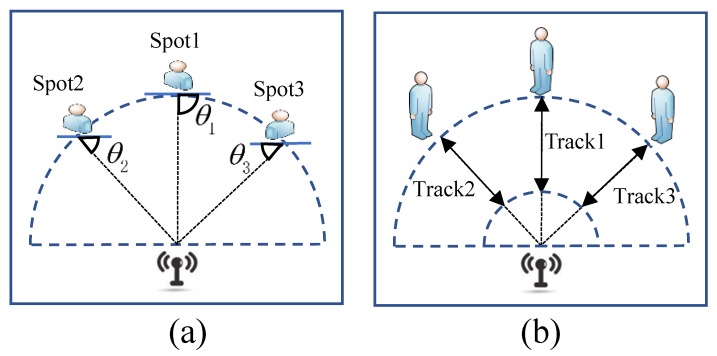
Experimental scenarios for static and moving targets. (**a**) Spot settings in static scenarios, and (**b**) track settings in moving scenarios.

The HEAR approach is implemented in the following settings:

1. The applied radar equipment is the NVA-R661 radar module, which is based on single chip CMOS NVA6201 impulse radar transceiver. It offers a spacial resolution of 1/256 m.
2. The deployed ECG acquisition module is based on the BMD101 chip and a bluetooth module BC147413. This ECG sensor is able to detect biological signals within μV to mV range. It provides information of real-time heart rate and respiration rate.
3. Experiments are carried out in resting scenarios and moving scenarios, as shown in Figure 8.
  In the resting scenario in Figure 8a, a tester stays static 1 m in front of the radar in three spots. The antenna-to-target angles $\theta_{1}$, $\theta_{2}$ and $\theta_{3}$ are respectively $\frac{\pi }{2}$, $-\frac{\pi }{4}$ and $\frac{\pi }{4}$.
  In the moving scenario in Figure 8b, a tester reciprocates on three tracks. Since it is difficult to assure a specific antenna-to-target angle during moving, the tester is merely required to face the radar in front in all tracks. Angles between tracks 1–3 to the radar correspond to $\frac{\pi }{2}$, $-\frac{\pi }{4}$ and $\frac{\pi }{4}$. To test the effectiveness in different distances, the track is respectively set within 1–2 m and 1.5–4.5 m. Each recording of activities lasts for 50 s.
4. The comparison experiment applies the STFT to acquire instantaneous frequencies and adopts VNCMD for one iteration to recover heartbeat waveforms. The HEAR approach employs VNCMD for 300 iterations with initial frequencies generated by STFT.

### 3.2. Results Evaluation

#### 3.2.1. Evaluations on Heart Rate Detections

(1) Resting scenario evaluation

**Figure 9:**
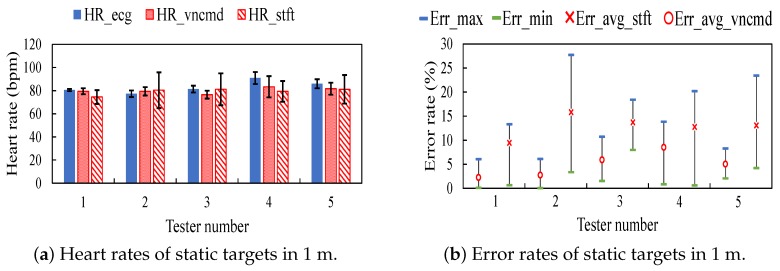
Results on static testers in spot 1.

Figure 9a shows heart rates measured on five resting testers in the spot1. The variable $HR_{ecg}$ is the reference heart rate detected in ECG recordings. Variables $HR_{vncmd}$ and $HR_{stft}$ are heart rates respectively extracted by HEAR and the scheme of STFT. Black lines illustrate the variance of measured and estimated heart rates. It observes that HEAR estimates heart rates with a variance smaller than that of the comparison scheme.

The error rate of heart rates is defined as ${Err}_{vncmd}=\frac{\left|HR_{vncmd}-HR_{ecg}\right|}{HR_{ecg}}$. Figure 9b shows error rates corresponding to Figure 9a. $Err_{max}$ is the maximum error and $Err_{min}$ is the minimum error. Err_avg_vncmd is the average error of HEAR and Err_avg_stft is the average error of STFT. It observes that the average error rate of HEAR is smaller than that of the comparison scheme.

**Figure 10:**
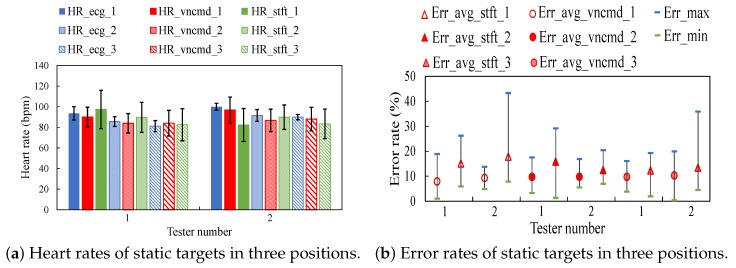
Results on static testers in spot 1–3.

Figure 10a illustrates heart rates tested in three spots 1–3. Figure 10b depicts error rates of heart rates corresponding to three spots. Variables HR_ecg_1, HR_ecg_2 and HR_ecg_3 are obtained on corresponding spots 1–3. It shows that error rates on different spots are similar in average values. For a specific resting tester, the error rate in spot 1 is slightly smaller than error rates in spots 2 and 3. The HEAR approach achieves a smaller average error rate compared to the scheme of STFT in three spots.

(2) Moving scenario evaluation

**Figure 11:**
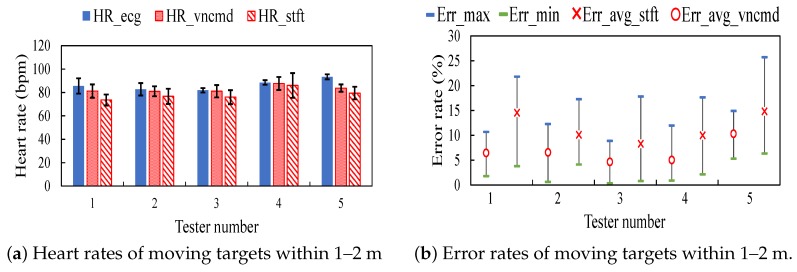
Results on moving testers in Track 1.

**Figure 12:**
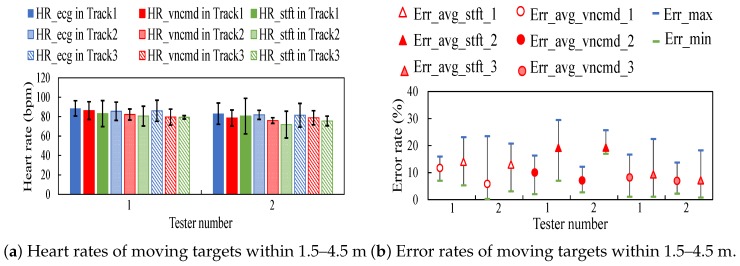
Results on moving testers in Tracks 1–3.

Figure 11a illustrates heart rates tested within a near distance 1–2 m in Track 1. Figure 11b depicts error rates of heart rates corresponding to Figure 11a. Figure 12a shows heart rates detected within a range 1.5–4.5 m in Tracks 1–3. Figure 12b displays error rates of heart rates corresponding to Figure 12a. From the comparison between error rates in near and long distances in Track 1, it observes the increments in error rates as the distance increases.

#### 3.2.2. Evaluations on HRV Analysis

Table 2 and Table 3 display discrepancies of parameters in HRV analysis compared with the reference ECG records. Specifically, SDNN is the standard deviation of R wave-to-R wave (RR) intervals, i.e., intervals between normal heartbeats. HF is the normalized power in the high frequency band 0.15–0.4 Hz and LF is the normalized power in the high frequency band 0.04–0.15 Hz. RMSSD is the root mean square of RR interval differences. The discrepancy of SDNN obtained by HEAR and ECG is calculated as $SDNN_{err\_vncmd}=\left|SDNN_{ecg}-SDNN_{vncmd}\right|$. The difference of SDNN derived by the scheme of STFT and ECG is computed in the same way. Similarly, deviations of RMSSD, LF and HF are acquired by the same computation. It is observed in Table 2 and Table 3 that the discrepancies of SDNN, LF and HF generated by HEAR are smaller than those obtained by the scheme of STFT. Compared to the scheme of STFT, the HEAR approach achieves a deviation of RMSSD smaller in the static scenario while larger in the moving state. It is attributed to interferences that large body movements introduce to the stableness of RR sequences in radar data.

#### 3.2.3. Evaluations on Bland–Altman Analysis

The difference of RR intervals in ECG and heartbeats is marked as RRd. The average of RRd is denoted as Bias. The discrepancy between Bias of VNCMD and the scheme of STFT is calculated as $\Delta \mathrm{Bias}=\left|Bias_{stft}\right|-\left|Bias_{vncmd}\right|$. The standard deviation of RRd is recorded as SD. The difference between SD of VNCMD and the comparison scheme is computed as $\Delta SD=\left|SD_{stft}\right|-\left|SD_{vncmd}\right|$.

Table 4 and Table 5 list parameters of the Bland–Altman analysis, respectively, on different static and moving testers. It proves the consistency that the value of Bias is close to zero. It shows that the HEAR approach is able to acquire beat-to-beat intervals consistent with reference RR intervals in ECG. The discrepancy between RR intervals in ECG and the scheme of STFT is decreased through applying the VNCMD.

**Figure 13:**
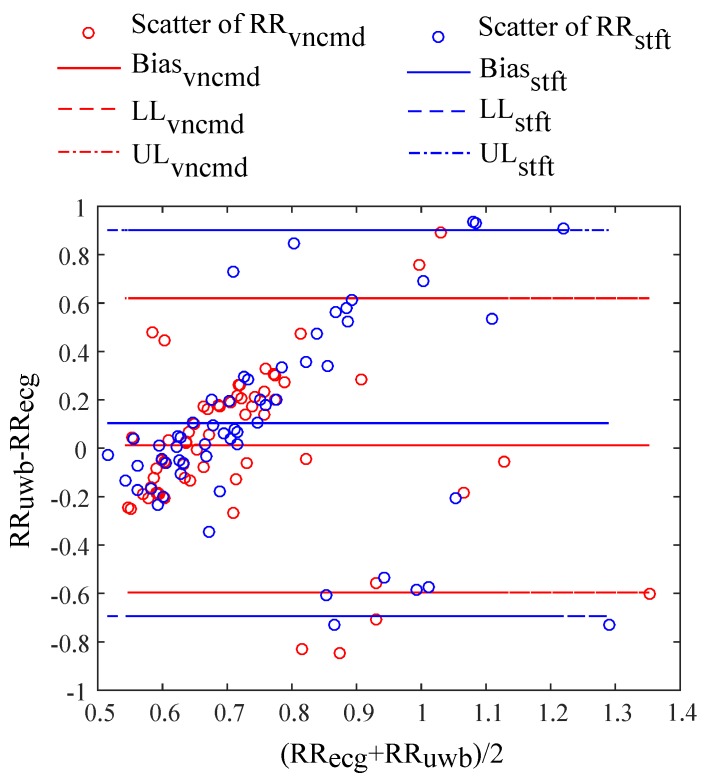
Scatters of R wave-to-R wave (RR) intervals in Bland–Altman analysis.

Figure 13 illustrates scatters of RR intervals in the Bland–Altman analysis. Scatters of RR intervals obtained by VNCMD are distributed more centrally within the upper limit UL and the lower limit LL that are respectively determined by Bias±1.96SD. It indicates that HEAR achieves a heartbeat signal whose RR intervals are more consistent with those of ECG, compared to heartbeats extracted by the scheme of STFT.

#### 3.2.4. Evaluations on HEAR Approach with Different Motion Frequencies

In order to analyze influences of motion frequencies to the HEAR approach, the tester is required to reciprocate within 1–2 m in different times. Experiments are repeated for seven times and evaluation parameters of heart rates and the HRV analysis are summarized in Table 6. In addition, the difference between real and estimated breathing times is calculated as well, denoted as ΔBR in Table 6. It shows that HEAR acquires an average error as 2.19 breathing times during 50 s. The motion frequency is equal to 0.16 Hz when the tester walks back or forth for eight times in 50 s. The average of error rates of heart rates ${Err}_{vncmd}$ is 6.15% and the fluctuation of ${Err}_{vncmd}$ shows no obvious relation with motion frequencies. In spite of fluctuations, $RMSSD_{err}$ and $SDNN_{err}$ take on a decreasing trend as the motion frequency increases.

To analyze the time profiling of HEAR, the running time of three procedures in HEAR is counted and averaged with different time lengths of input radar data. Table 7 shows time consumptions of procedures for the wavelet-based filtering, the body movement compensation and the VNCMD-based estimation. Specifically, the input radar data which lasts for 5 s contains 200 echos, since the time sampling rate is 40 Hz. In this case, for a specific echo, the time consumption of wavelet-based filtering is 45.85 ms. Although the time consumption of the VNCMD-based estimation increases as the length of input data arises, its values are smaller than 1 s.

To compare the heart rates estimated by VNCMD with and without optimizations in initial frequencies, results of comparison experiments are listed in Table 8. The variable ${Err}_{original}$ denotes the error rate between heart rates obtained by ECG and the original VNCMD method without optimizing initial frequencies. ${Err}_{stft}$ is the error rate of heart rates introduced by initial frequencies determined by the STFT method. ${Err}_{vncmd}$ is acquired with optimized initial frequencies. From the comparison between ${Err}_{stft}$ and ${Err}_{original}$ in Table 8, it indicates that the original VNCMD method fails to reduce error rates with unoptimized initial frequencies.

## 4. Conclusions

This paper proposed a Heartbeat Estimation And Recovery approach to extract instantaneous heartbeat signals using an IR-UWB radar. To remove static clutter, HEAR modifies the wavelet-based methods with a two-parameter exponential threshold, according to characteristics of amplitudes attenuations. The HEAR approach builds a mapping from maximum echo amplitudes to the fast time delay to acquire a chirp signal which contains body micromovements and large body movements. Based on this mapping, it is feasible to cancel large body movements estimated by the extended Kalman filter through a simple subtraction. Then, HEAR adopts the VNCMD to obtain instantaneous frequencies and waveforms of heartbeats with optimizing initial modal frequencies. Results of the HRV analysis and the Bland–Altman analysis validate that HEAR captures instantaneous heartbeat signals of moving targets by effectively compensating large body movements.

## Figures and Tables

**Table 1:** Comparisons on reconstruction errors of six kinds of wavelets with impulse radio-ultra wideband radar data.

	**bior2.4**	**bior2.6**	**Haar**	**db2**	**db6**
$Err_{max}$	$2.7756\times 10^{-16}$	$2.7756\times 10^{-16}$	$2.2204\times 10^{-16}$	$9.2937\times 10^{-13}$	$8.5720\times 10^{-13}$
$Err_{avg}$	$1.1118\times 10^{-18}$	$1.1365\times 10^{-18}$	$1.3234\times 10^{-18}$	$7.5501\times 10^{-15}$	$1.0843\times 10^{-14}$
	**sym8**	**sym5**	**coif1**	**coif3**	**meyr**
$Err_{max}$	$1.9307\times 10^{-13}$	$2.2660\times 10^{-13}$	$1.6060\times 10^{-12}$	$1.1326\times 10^{-12}$	$1.4447\times 10^{-6}$
$Err_{avg}$	$1.9147\times 10^{-15}$	$2.2487\times 10^{-15}$	$1.2080\times 10^{-14}$	$1.4078\times 10^{-14}$	$2.4692\times 10^{-8}$

**Table 2:** Heart rate variability analysis on resting testers in Spot 1.

	Tester 1	Tester 2	Tester 3	Tester 4
$SDNN_{err\_vncmd}$ (ms)	117.99	99.92	116.02	138.71
$SDNN_{err\_stft}$ (ms)	186.33	118.21	179.38	202.88
$RMSSD_{err\_vncmd}$ (ms)	136	131.23	101.52	129.99
$RMSSD_{err\_stft}$ (ms)	204.93	157.21	151.34	191.86
$LF_{err\_vncmd}$ (${\mathrm{ms}}^{2}$)	842	892	838	1077
$LF_{err\_stft}$ (${\mathrm{ms}}^{2}$)	21,041	13,649	13,715	16,500
$HF_{err\_vncmd}$ (${\mathrm{ms}}^{2}$)	2102	2676	705	2242
$HF_{err\_stft}$ (${\mathrm{ms}}^{2}$)	50,863	33,294	30,517	40,397

**Table 3:** Heart rate variability analysis on on moving testers in Track 1.

	Tester 1	Tester 2	Tester 3	Tester 4
$SDNN_{err\_vncmd}$ (ms)	73.18	169.65	74.09	169.7
$SDNN_{err\_stft}$ (ms)	96.37	194.58	83.05	185.5
$RMSSD_{err\_vncmd}$ (ms)	43.49	144.34	137.36	162.1
$RMSSD_{err\_stft}$ (ms)	36.41	137.72	156.34	130.3
$LF_{err\_vncmd}$ (${\mathrm{ms}}^{2}$)	1787	1837	2316	1549
$LF_{err\_stft}$ (${\mathrm{ms}}^{2}$)	14,497	15,045	10,654	10,950
$HF_{err\_vncmd}$ (${\mathrm{ms}}^{2}$)	3788	2629	4672	1700
$HF_{err\_stft}$ (${\mathrm{ms}}^{2}$)	29,747	33,061	23,093	23,476

**Table 4:** Bland–Altman analysis on resting testers in Spot 1.

	Tester 1	Tester 2	Tester 3	Tester 4
ΔBias (ms)	15.15	40.77	35.75	72.43
ΔSD (ms)	93.71	37.4	29.97	1.59

**Table 5:** Bland–Altman analysis on moving testers in Track 1.

	Tester 1	Tester 2	Tester 3	Tester 4
ΔBias (ms)	25.51	10.23	21.09	2.52
ΔSD (ms)	26.58	29.35	36.33	18.77

**Table 6:** Respiration rate and heart rate estimation and HRV analysis with different motion frequencies.

Parameters	Test 1	Test 2	Test 3	Test 4	Test 5	Test 6	Test 7	Average
motion frequency (Hz)	0.16	0.22	0.29	0.41	0.5	0.77	0.8	-
maximum speed (m/s)	1.01	1.35	1.24	1.28	1.8	1.79	2.82	-
ΔBR	4.64	0.34	1.67	2.42	0.61	4.65	1.07	2.19
${Err}_{vncmd}\left(\%\right)$	7.07	6.02	0.54	12.39	8.82	0.47	7.73	6.15
$SDNN_{err\_vncmd}$ (ms)	66.4	134.7	15.7	127.2	16.1	124.9	47.3	76
$RMSSD_{err\_vncmd}$ (ms)	150.9	33.6	34.5	132.6	93	168.4	23.9	91
$LF_{err\_vncmd}$ (${\mathrm{ms}}^{2}$)	11,712	1167	2997	2502	1387	808	746	3045
$HF_{err\_vncmd}$ (${\mathrm{ms}}^{2}$)	7282	5855	9939	1839	1815	924	107	3966

**Table 7:** Time profiling of Procedure 1 for wavelet-based filtering, Procedure 2 for body movement compensation and Procedure 3 for variational nonlinear chirp mode decomposition based estimation in Heartbeat Estimation And Recovery approach.

	Test 1	Test 2	Test 3	Test 4
Input data length (s)	5	10	15	20
Procedure 1 (s)	9.17	20.14	31.6	45.5
Procedure 2 (ms)	0.9	0.9	1.1	1.5
Procedure 3 (ms)	150.7	262.3	193.6	392.6
Total time per echo (ms)	46.61	51	52.99	57.37

**Table 8:** Comparisons on estimated heart rates with and without optimizations in initial frequencies of VNCMD.

	Test 1	Test 2	Test 3	Test 4	Test 5
${Err}_{vncmd}\left(\%\right)$	1.82	3.79	10.35	7.7	6.76
${Err}_{stft}\left(\%\right)$	7.07	17.05	18.92	17.78	13.91
${Err}_{original}\left(\%\right)$	13.38	21.49	20.89	18.59	19.48
